# ELISA Development for Serum Hemeoxygenase-1 and Its Application to Patients with Acute Respiratory Distress Syndrome

**DOI:** 10.1155/2018/9627420

**Published:** 2018-04-18

**Authors:** Yu Hara, Masaharu Shinkai, Masataka Taguri, Kenjiro Nagai, Satoru Hashimoto, Takeshi Kaneko

**Affiliations:** ^1^Department of Pulmonology, Yokohama City University Graduate School of Medicine, Yokohama, Japan; ^2^Department of Biostatistics, Yokohama City University School of Medicine, Yokohama, Japan; ^3^Division of Intensive Care Unit, Kyoto Prefectural University of Medicine, Kyoto, Japan

## Abstract

**Background:**

Hemeoxygenase-1 (HO-1) is an essential enzyme in heme catabolism and has been proposed as a biomarker of lung disease prognosis. We modified a commercial HO-1 enzyme-linked immunosorbent assay (ELISA) kit to achieve higher sensitivity and evaluated if serum HO-1 could be a biomarker to predict the prognosis of acute respiratory distress syndrome (ARDS) patients.

**Methods:**

Serum samples were collected from 15 healthy volunteers to validate the modified ELISA. In the 22 patients with ARDS who were enrolled, serum HO-1 was measured upon diagnosis (D0) and at 7 days after diagnosis (D7).

**Results:**

The serum HO-1 concentration could be measured in all healthy volunteers. The intra- and interassay tests and the percentage recovery test were acceptable. Compared with normal control subjects, patients with ARDS had significantly higher D0 HO-1 concentrations (75.4 ng/mL versus 31.7 ng/mL, *P* < 0.001). The 28-day survival was significantly better in patients with low D0 HO-1 (<75.8 ng/mL) than in those with high D0 HO-1 (≥75.8 ng/mL) (mortality rate: 18% versus 73%, *P*=0.016). Nonsurvivors had significantly higher D0 and D7 HO-1 concentrations than survivors (*P* < 0.05).

**Conclusion:**

Serum HO-1 may be a useful biomarker to predict the prognosis of patients with ARDS.

## 1. Introduction

Hemeoxygenase-1 (HO-1) is a 32 kDa heat shock protein that converts heme to carbon monoxide, iron, and bilirubin [1–3]. HO-1 is released from alveolar macrophages, bronchial epithelial cells, and inflammatory cells after stimulation by cytokines, hypoxia, exogenous nitric oxide, or diesel exhaust particles [4, 5]. Its expression in lung tissue was reported to increase in patients with diffuse alveolar damage (DAD), desquamative interstitial pneumonia, pulmonary sarcoidosis, and silicosis, whereas its expression decreased in patients with usual interstitial pneumonia (UIP) and chronic pulmonary obstructive disease (COPD) [5–9]. Peripheral blood HO-1 measured by enzyme-linked immunosorbent assay (ELISA) was reported to be useful in monitoring disease activity in patients with hemophagocytic syndrome and adult-onset Still's disease and is a predictor of lung function decline in patients with silicosis [10, 11]. However, there is no commercial detection kit to accurately measure serum HO-1 because the previously used kit was designed to measure HO-1 in cell lysates and tissue extracts and can be affected by factors contained in serum [11]. Therefore, we developed a new method of serum HO-1 measurement using an optimized assay buffer and validated the sensitivity of this ELISA technique of serum HO-1 measurement in predicting the clinical outcomes in patients with acute respiratory distress syndrome (ARDS).

## 2. Materials and Methods

### 2.1. Study Subjects for the Researches on Assay Validation and ARDS

For the research on assay validation, we recruited 15 healthy volunteers (mean age, 34.9 ± 8.6 years) from the medical staff of the Respiratory Disease Center, Yokohama City University Medical Center. For the research on ARDS, 22 patients who met the Berlin definition for ARDS [12] during ICU stay were recruited from April 2011 to March 2014. Normal control subjects comprised healthy adults who were admitted to the National Defense Medical College Hospital for health exam.

### 2.2. Serum HO-1 ELISA

Serum HO-1 concentrations were measured using the HO-1 ELISA kit (Enzo, Farmingdale, NY, USA) and the ImmunoSet^™^ HO-1 ELISA development set (Enzo, Farmingdale, NY, USA), according to the manufacturers' instructions. The former kit used undiluted serum samples or samples diluted at 1 : 2 (commercial ELISA), whereas the latter kit used a modified assay buffer and serum samples diluted at 1 : 20 (modified ELISA).

### 2.3. Buffer Formulations and Reagent for the Modified ELISA

The buffer formulations used were coating buffer (phosphate-buffered saline (PBS)); blocking buffer (PBS containing 1% bovine serum albumin (BSA) and 0.05% Tween 20); assay buffer (PBS containing 1% BSA, 0.05% Tween 20, 0.1 M NaCl, 5 mM ethylenediaminetetraacetic acid (EDTA), and 50 *μ*g/mL mouse IgG); wash buffer (PBS containing 0.1% *v*/*v* Tween 20); and 2% mouse serum added to the standard diluents. The reagents were ImmunoSet HO-1 ELISA development set (Cat#ADI-960-800, Enzo, Farmingdale, NY, USA); 3, 3′, 5, 5′ tetramethylbenzidine solution (Cat#T0440); stop solution (Cat#S5814); IgG from mouse serum (Cat#I5381); mouse serum (#CatM5905); BSA fraction V (Cat#A9647, Sigma, St. Louis, MO, USA); 10 × PBS buffer (pH 7.4, Wako Pure Chemical Industries, Ltd., Chuo-ku, Osaka, Japan); Ultra-Pure Tween 20 (Cat#EC-607, National Diagnostics, Atlanta, Georgia, USA); Nunc Immunoplate MaxiSorp 96-well ELISA plates (#Cat439454, Thermo Fisher Scientific Inc., Waltham, MA, USA); and ImmunoMini NJ-2300 Microplate reader (BIOTEC CO., Ltd., Tokyo, Japan).

### 2.4. Data Collection

The extracted data included age, gender, body mass index, and ARDS severity and etiology. The acute physiology and chronic health evaluation (APACHE) II was recorded during ICU visit [13]. The outcomes including the duration of mechanical ventilation and length of ICU stay were recorded.

We divided the patients into the survivors group (survived for >28 days) and the nonsurvivors group (survived for ≤28 days). Each patient was evaluated for peripheral blood HO-1, lactate dehydrogenase (normal < 225 U/L), C-reactive protein (normal < 0.3 mg/dL); PaO_2_/F_I_O_2_; and lung injury score at the time of diagnosis, which was designated as D0, and at 7 days after diagnosis, which was designated as D7. The lung injury score (Murray score), which is a commonly utilized measure of lung injury severity, is calculated by adding the chest radiograph score (0–4), positive end-expiratory pressure score (0–4), hypoxemia score (0–4), and respiratory system compliance score (0–4) and dividing the sum by the number of components; a Murray score of 0 indicates no lung injury, and a score of >2.5 indicates severe lung injury [14]. ΔHO-1 was defined as D7 serum HO-1 concentration minus D0 serum HO-1 concentration.

### 2.5. Statistical Analysis

Data were expressed as median with 25th to 75th percentiles or mean ± standard deviation (SD). We compared the results between the commercial ELISA and the modified ELISA using Assay Blaster® software (Enzo, Farmingdale, NY, USA). Statistical analysis was performed using JMP10 and SAS version 9.3 (SAS Institute Inc., North Carolina, USA). Group comparisons were made using Wilcoxon's rank-sum test or chi-square test, as appropriate. Spearman correlation coefficients were calculated to assess the relationship between serum HO-1 and APACHE II score. The applicability of serum HO-1 in predicting survival was evaluated using the area under a receiver operating characteristic (ROC) curve; the sensitivity and specificity were calculated at several cutoff points. Survival curves were generated using the Kaplan–Meier method and were compared using the log-rank test. A *P* value of <0.05 was considered significant.

### 2.6. Study Approval

All aspects of this research were approved by the institutional review board of Yokohama City University Medical Center (approval number: D1303019) and the National Defense Medical College (approval number: 913). In the research on ARDS, the severely ill condition or deep sedation of the patients precluded us from obtaining informed consent from the patients themselves. Therefore, informed consent was obtained from the patients' relatives or their legal guardians instead. The other subjects of this research, including the normal control, provided informed consent prior to participation in this research.

## 3. Results

### 3.1. The Research on Assay Validation

#### 3.1.1. Detecting Serum HO-1 Using the Commercial ELISA

**Figure 1:**
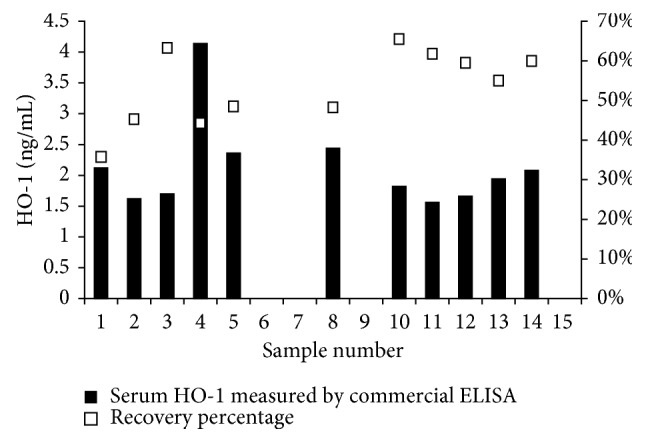
Measurement of serum HO-1 in healthy volunteers using a commercial ELISA kit (HO-1 ELISA kit; Enzo, Farmingdale, NY, USA). Serum HO-1 at 1 : 1 dilution (×1) is plotted against the % recovery, which was calculated by spiking the samples with 3.13 ng/mL of the recombinant human HO-1 protein at a dilution of 1 : 2. ELISA, enzyme-linked immunosorbent assay; HO-1, hemeoxygenase-1.

The serum interference in detecting HO-1 was measured by the spike and recovery test. Using the commercial ELISA kit, we found a serum interference of 63% ± 17% (range, 39%–93%) in 11 samples (Figure 1). The ImmunoSet ELISA development set without modified buffers was also tested, and the interference with HO-1 detections was 48% ± 19% (range, 21%–80%) in 10 samples.

#### 3.1.2. Assay Validation for the Modified ELISA

**Figure 2:**
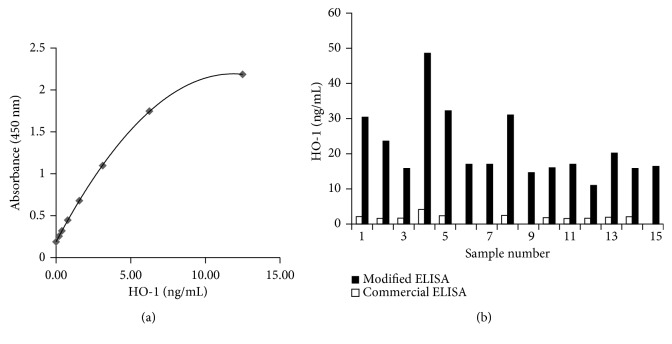
Validation of serum HO-1 ELISA. (a) In a typical standard curve using modified ELISA, the correlation coefficient from 7 standard curves was 0.998 ± 0.002. The lower limit of detection was 0.038 ng/mL. (b) Comparison shows that the average serum HO-1 of 15 healthy samples was 17.2 ± 9.5 ng/mL by modified ELISA (all with measurable concentrations) and 2.1 ± 0.7 ng/mL by commercial ELISA (undetectable concentrations in 4 of 15 samples). ELISA, enzyme-linked immunosorbent assay; HO-1, hemeoxygenase-1.

The modified ELISA was validated for its performance in detecting serum HO-1. A typical ELISA standard curve for HO-1 is shown in Figure 2(a). Seven calibration standards that were prepared using the modified assay buffer with 2% mouse serum were analyzed using a four-parameter logistic regression algorithm to fit the optical density as a function of the HO-1 concentration. The mean correlation coefficient from these 7 standard curves was 0.998 ± 0.002. The lower limit of detection was determined to be 0.038 ng/mL by interpolation at 2 SD above the mean background signal. Intra-assay precision was tested in 1 run using 6 samples in 10 replicates, whereas interassay precision was tested in 6 separate runs, twice daily, using 6 samples in duplicates. The average coefficient intra-assay and interassay variations were 7% (serum HO-1 concentration, 36.3 ± 1.6 ng/mL) and 10% (serum HO-1 concentration, 32.5 ± 3.0 ng/mL), respectively (Table 1). The possible interference by other compounds in measuring serum HO-1 was investigated by spiking the serum with known concentrations of purified HO-1 (1, 5, and 10 ng/mL) and comparing the measured values with the expected values; we found an average recovery of 98%, 105%, and 104% respectively, with less than 10% coefficient of variation (Table 2).

#### 3.1.3. Comparison of the Modified ELISA and the Commercial ELISA

Detection of serum HO-1 was compared between the commercial ELISA and the modified ELISA using serum from 15 healthy volunteers (Figure 2(b)). The modified ELISA yielded higher HO-1 concentrations than the commercial ELISA (17.2 ng/mL ± 9.5 ng/mL versus 2.1 ng/mL ± 0.7 ng/mL). The modified ELISA detected HO-1 in all samples, whereas the commercial ELISA did not detect HO-1 in 4 samples.

### 3.2. The Research on ARDS

#### 3.2.1. Comparison of Serum HO-1 Concentrations between Patients with ARDS and Normal Control Subjects

**Figure 3:**
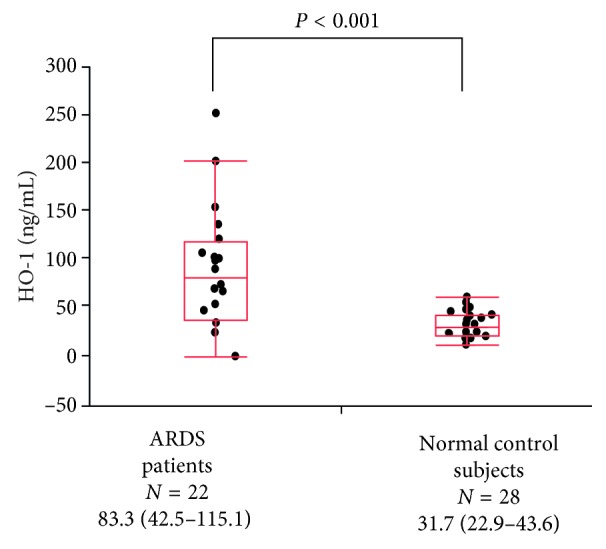
Comparison of serum HO-1 concentrations between patients with ARDS and normal control subjects. The patients with ARDS had significantly higher D0 HO-1 concentrations than the normal control subjects (75.4 ng/mL versus 31.7 ng/mL, *P* < 0.001). The center bold line is the median value; the bottom and top of the boxes represent the 25th to 75th percentiles, respectively; and the whiskers are 95% confident intervals. ARDS, acute respiratory distress syndrome; HO-1, hemeoxygenase-1.

The clinical characteristics of the 22 patients with ARDS, including 12 survivors and 10 nonsurvivors, are summarized in Table 3. The patients with ARDS had significantly higher D0 HO-1 concentrations than the normal control subjects (75.4 ng/mL versus 31.7 ng/mL, *P* < 0.001) (Figure 3).

#### 3.2.2. Survival in Patients with ARDS

**Figure 4:**
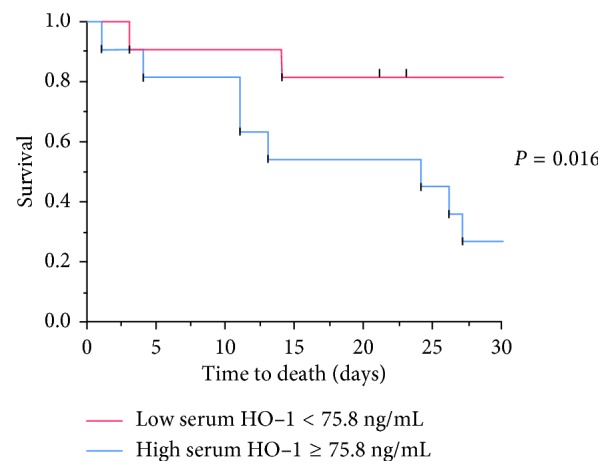
Comparison of patients according to serum HO-1 concentrations. The 28-day survival was significantly better in the low serum HO-1 group (*N*=11) than in the high serum HO-1 group (*N*=11) (*P*=0.016). HO-1, hemeoxygenase-1.

In the evaluation of the applicability of serum HO-1 in predicting survival, the area under the ROC curve was 0.76. The best cutoff concentration of serum HO-1 for estimating 28-day survival was 75.8 ng/mL, with sensitivity of 72% and specificity of 73%. Using this cutoff concentration of 75.8 ng/mL, the 22 patients with ARDS were divided into 2 groups, the low serum HO-1 group (*N*=11) and the high serum HO-1 group (*N*=11). The Kaplan–Meier survival curves of the 2 groups were significantly different on the log-rank test (*P*=0.016) (Figure 4). The mortality rate was 18% for the former group and 73% for the latter group.

#### 3.2.3. Correlations between ΔHO-1/D0 HO-1 and the Clinical Parameters

**Figure 5:**
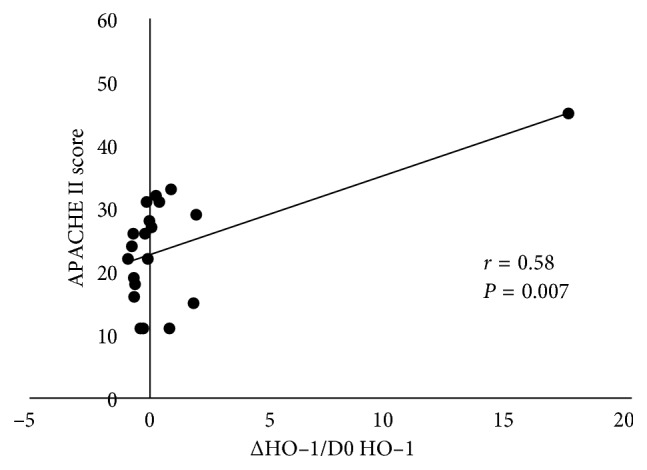
Correlations between ΔHO-1/D0 HO-1 and the clinical parameters. ΔHO-1/D0 HO-1 is significantly correlated with the APACHE II score. D0 HO-1 = HO-1 concentration measured upon diagnosis. D7 HO-1 = HO-1 concentration measured at 7 days after diagnosis. ΔHO-1 = D7 HO-1 minus D0 HO-1. APACHE, acute physiology and chronic health evaluation; D0, day 0; D7, day 7; HO-1, hemeoxygenase-1; ICU, intensive care unit.

Two of the 10 nonsurvivors died within 7 days; therefore, we calculated the values of ΔHO-1/D0 HO-1 for 12 survivors and 8 nonsurvivors. As shown in Figure 5, ΔHO-1/D0 HO-1 was significantly correlated with the APACHE II score (*r*=0.58,  *P*=0.007). The patient with the outlier on the top-right of Figure 5 was a survivor and this patient had 17.4 of ΔHO-1/D0 HO-1 and 45 of the APACHE II score. Also, the duration of mechanical ventilation was 49 days which was the longest duration of all enrolled patients.

#### 3.2.4. Comparison of Serum HO-1 Concentrations at D0 and D7 between Nonsurvivors and Survivors

**Figure 6:**
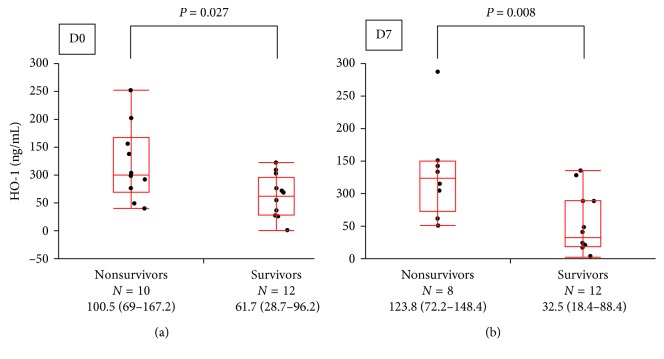
Comparison of serum HO-1 concentrations according to survival. The HO-1 concentrations were significantly higher in nonsurvivors than in survivors on day 0 (100.5 ng/mL versus 61.7 ng/mL, *P*=0.027) and day 7 (123.8 ng/mL versus 32.5 ng/mL, *P*=0.008). The center bold line is the median value; the bottom and top of the boxes represent the 25th to 75th percentiles, respectively; and the whiskers are 95% confident intervals. HO-1, hemeoxygenase-1.

As shown in Figure 6, the serum HO-1 concentration was significantly higher in nonsurvivors than in survivors on D0 (100.5 ng/mL versus 61.7 ng/mL, *P*=0.027) and on D7 (123.8 ng/mL versus 32.5 ng/mL, *P*=0.008).

## 4. Discussion

The HO-1 ELISA kit (Enzo, Farmingdale, NY), which was designed to measure HO-1 in cell lysates and tissue extracts, had low sensitivity for detecting serum HO-1. Although serum HO-1 measured by the HO-1 ELISA kit has been previously reported to be a predictor of lung function decline in patients with silicosis [11], this method could not detect serum HO-1 in 4 of the 15 healthy volunteers in this study (Figure 2(b)). Moreover, we estimated a relatively difficult detection of serum HO-1 in patients with UIP and COPD, in which lung tissue HO-1 was reported to decrease [5, 9]. Therefore, we attempted to improve the sensitivity of serum HO-1 detection by including mouse *γ*-globulin in the assay buffer, changing the concentration of NaCl in the buffer, and adding EDTA to decrease the detection of nonspecific serum proteins and found that a buffer containing 50 *μ*g/mL mouse IgG, 0.1 M NaCl, and 5 mM EDTA with 5% serum was the best buffer in decreasing serum interference in the assay [15–19]. In this research, we measured serum HO-1 concentrations in patients with ARDS using the modified ELISA and evaluated the correlations between serum HO-1 concentrations and clinical outcomes. Considering low sensitivity and instability of recovery percentage of the commercial ELISA to detect serum HO-1, we did not try to measure serum HO-1 of patients with ARDS using the commercial ELISA.

In patients with DAD which is the typical pathological pattern of ARDS, HO-1 was reported to be overexpressed in lung tissues, including alveolar macrophages, epithelial cells, endothelial cells, and fibroblasts, reflecting a pulmonary cellular protective reaction [6]. In our preliminary data, we found that the arterial concentration of carboxyhemoglobin, which is the breakdown product of HO-1, was significantly higher in patients with acute exacerbation of idiopathic pulmonary fibrosis (IPF) (pathological DAD) than in patients with stable IPF [20, 21]. In the present study, the serum HO-1 concentration of patients with ARDS was significantly higher than that of normal control subjects. Therefore, we believe that serum HO-1 measurement is useful for detecting ARDS patients in the clinical setting.

The APACHE II score is frequently used to measure disease severity in the ICU, but has several limitations in predicting clinical outcome [13]. In the present study, we demonstrated significant correlations of ΔHO-1/D0 HO-1 with the APACHE II score. This result indicates that when the APACHE II score is high, serum HO-1 concentration increases from D0 to D7. Furthermore, we found that although the APACHE II score did not significantly differ between survivors and nonsurvivors, the serum HO-1 concentration of nonsurvivors was higher than that of survivors. Therefore, it may be important to follow up the D0 and D7 serum HO-1 concentration and this measurement may be more sensitive than the APACHE II score in predicting the outcome of patients with ARDS. However, further studies involving a large number of critically ill patients at risk for ARDS are needed to verify this hypothesis.

HO-1 induction is thought to represent a protective response to oxidative stress, which correlates with poor clinical outcome when persistent [1, 22]. In a previous report, we found that decreases in HO-1 concentrations in serum and bronchoalveolar lavage fluid reflected clinical improvement in patients with acute fibrinous and organizing pneumonia [23]. In another clinical research on patients with inflammatory pulmonary diseases, the concentrations of arterial carboxyhemoglobin decreased after treatment [24]. In this present study, we demonstrated that both D0 and D7 HO-1 concentration were significantly higher in nonsurvivors than in survivors. Therefore, we speculated that a high serum HO-1 concentration of both D0 and D7 reflects stronger oxidative stress and leads to poor clinical outcomes.

This study had several limitations. First, because we established a more sensitive ELISA method for serum HO-1 measurement than the commercial ELISA and validated the sensitivity of this new ELISA technique in patients with ARDS with high HO-1 production, we should examine the ability to detect serum HO-1 in patients with diseases with low HO-1 production, including COPD, UIP, and so on. In our preliminary data, we succeeded in detecting serum HO-1 in 40 of COPD patients (all patients with COPD were measurable and serum HO-1 concentration was 23.8 ± 11.9 ng/mL (a detailed data not shown)). Second, only univariate analysis could be performed because of the small number of patients with ARDS. It is important to do this study on a larger number of patients in the future. Third, the cause of ARDS in this population was heterogeneous; future studies should evaluate the clinical utility of serum HO-1 according to the cause of ARDS. Fourth, we could not analyze the correlation between serum HO-1 and the other previously established biomarkers of ARDS in this study.

## 5. Conclusions

The modified ELISA technique that we established for detecting serum HO-1 appeared to be more sensitive than the commercial ELISA used in previous publications. Serum HO-1 measurement can be useful for detecting ARDS patients. In addition, measurement of baseline and serial serum HO-1 concentrations is important in predicting the prognosis of patients with ARDS.

## Figures and Tables

**Table 1:** Intra- and interassay test.

*N*=6	Serum HO-1 (ng/mL)	% coefficient variations (%)
Intra-assay test	36.3 ± 1.6	7
Interassay test	32.5 ± 3.0	10

Values are reported as mean ± SD or number (%). The acceptable range of coefficient variation is less than 10%. HO-1, hemeoxygenase-1.

**Table 2:** Evaluation of influence of serum interferences using the spike and recovery test.

*N*=13	Serum HO-1 (ng/mL)	% coefficient variations (%)^b^
Low, 1 ng/mL^a^	98	10
Middle, 5 ng/mL^a^	105	8
High, 10 ng/mL^a^	104	9

^a^Low, middle, and high mean known concentrations of purified HO-1; ^b^the acceptable range of coefficient variation is less than 10%. HO-1, hemeoxygenase-1.

**Table 3:** Clinical characteristics of patients with ARDS.

Characteristics	Overall cases (*N*=22)	Survivors (*N*=12)	Nonsurvivors (*N*=10)	*P* values, survivors versus nonsurvivors
Age, years	68.5 (62.0–75.0)	68.5 (56.5–75.0)	67.5 (63.0–76.0)	0.741
Male sex, *N* (%)	16 (73)	6 (50)	10 (100)	0.009
Body mass index	21.1 (18.6–23.1)	21.5 (20.3–23.4)	20.5 (18.3–23.1)	0.410
APACHE II score	25.5 (18.0–31.0)	23.0 (17.0–31.0)	26.5 (19.0–29.0)	0.644
Severity of ARDS, *N* (%)				0.548
Mild	7 (32)	5 (42)	2 (20)	
Moderate	11 (50)	5 (42)	6 (60)	
Severe	4 (18)	2 (17)	2 (20)	
Etiology of ARDS, *N* (%)				0.145
Infection	14 (64)	6 (42)	8 (80)	
Other causes	8 (36)	6 (58)	2 (20)	
Respiratory parameters				
D0 PaO_2_/F_I_O_2_ ratio	165 (118–262)	176 (131–302)	141.5 (105.0–185.0)	0.291
D7 PaO_2_/F_I_O_2_ ratio	198 (123–280)	260 (183–365)	140.5 (97.5–212.5)	0.019
D0 LIS	2.5 (2.0–2.8)	2.3 (1.6–2.5)	2.8 (2.5–3.5)	0.015
D7 LIS	2.3 (1.8–2.8)	2.0 (1.5–2.3)	3.0 (2.5–3.3)	0.008
Blood parameters				
D0 HO-1, ng/mL	75.4 (39.1–111.6)	61.7 (28.7–96.2)	100.5 (69.0–167.2)	0.027
D7 HO-1, ng/mL	74.5 (22.1–130.6)	32.5 (18.4–88.4)	123.8 (72.2–148.4)	0.008
D0 LDH, U/L	307 (242–437)	307 (253–448)	305 (237–437)	0.843
D7 LDH, U/L	326 (263–448)	326 (270–473)	329 (245–418)	0.847
D0 CRP, mg/dL	14.8 (9.7–24.1)	12.8 (9.2–26.6)	16.5 (11.4–20.3)	0.921
D7 CRP, mg/dL	6.9 (2.5–13.8)	7.7 (1.9–13.6)	5.7 (4.9–16.6)	0.642
Outcomes				
Mechanical ventilation, days	11.5 (7.0–21.0)	9.5 (7.5–17.0)	14.0 (5.0–24.0)	0.668
Length ICU stay, days	13.0 (9.0–26.0)	11.5 (10.0–27.0)	14.0 (5.0–26.0)	0.741

Values are reported as median with 25–75th percentiles or %, unless otherwise indicated. Two of the 10 nonsurvivors died within 7 days. APACHE, acute physiology and chronic health evaluation; ARDS, acute respiratory distress syndrome; LIS, lung injury score; HO-1, hemeoxygenase-1; LDH, lactate dehydrogenase; CRP, C-reactive protein.

## Abbreviations

APACHE: Acute physiology and chronic health evaluation
ARDS: Acute respiratory distress syndrome
BSA: Bovine serum albumin
COPD: Chronic pulmonary obstructive disease
DAD: Diffuse alveolar damage
EDTA: Ethylenediaminetetraacetic acid
ELISA: Enzyme-linked immunosorbent assay
HO-1: Hemeoxygenase-1
IPF: Idiopathic pulmonary fibrosis
PBS: Phosphate-buffered saline
ROC: Receiver operating characteristic
SD: Standard deviation.
